# Rationale, Design, and Profiles of the New Integrated Suburban Seniority Investigation (NISSIN) Project: A Study of an Age-Specific, Community-Based Cohort of Japanese Elderly

**DOI:** 10.2188/jea.JE20081026

**Published:** 2009-09-05

**Authors:** Tetsuhisa Kitamura, Takashi Kawamura, Akiko Tamakoshi, Kenji Wakai, Masahiko Ando, Yoshiyuki Ohno

**Affiliations:** 1 Kyoto University Health Service, Kyoto, Japan; 2 Department of Public Health, Aichi Medical University School of Medicine, Nagakute, Aichi, Japan; 3 Department of Preventive Medicine/Biostatistics and Medical Decision Making, Nagoya University Graduate School of Medicine, Nagoya, Japan

**Keywords:** cohort study, elderly, age-specific, physical functioning, quality of life

## Abstract

**Background:**

Although there have been many studies on aging in a number of developed countries, data on the effects of aging during early senescence are scarce. We designed a study to investigate an age-specific cohort in a suburban Japanese city to determine the factors that contribute to living long and well.

**Methods:**

In every year from 1996 through 2005, residents of Nissin City, Japan who were about to reach the age of 65 years participated in health check-ups and completed a baseline self-administered questionnaire that included items on demographic and lifestyle characteristics, physical function, and quality of life. When the participants reached 70 years of age, they underwent secondary health check-ups at the same site, or received home visits from public health nurses, and their health-related outcomes were noted.

**Results:**

A total of 3073 64-year-olds were enrolled in the study (response rate, 43.9%). There was considerable intersexual variation in demographic and lifestyle factors. Among men and women, 24.3% and 3.0% were current smokers, respectively, and 68.7% and 19.5% were current alcohol drinkers. Cohort members were in slightly better physical condition than the Japanese general population: they were less likely to be obese and hypertensive and more likely to have 20 teeth or more. Follow-up of the cohort is ongoing.

**Conclusions:**

We have established a unique age-specific cohort with a consecutive entry–exit system. This project should provide data on early changes in health and related factors in this new era of longevity.

## INTRODUCTION

In Japan, many people live to an advanced age.^1^ The proportion of people aged 65 years or older has continuously increased, from 4.9% in 1950 to 21.5% in 2007, and it is expected to reach 35.7% in 2050.^2^ However, the increasing number of elderly people suffering from dementia and depression is becoming a serious problem. Dementia is associated with physical activity^3^ and diet,^4^ and depression in elders appears to increase mortality^5^ and cognitive deterioration.^6^ Therefore, a positive mental state is a key factor in longevity.

A cohort study is an effective epidemiological method in investigating the association between lifestyle and health events. A number of moderately sized cohort studies have been conducted to evaluate mortality/morbidity and related factors among elderly people, both in Japan^7^^–^^12^ and worldwide.^13^^–^^18^ Most participants in such cohorts were old-old, among whom life-threatening events are frequent. However, preclinical changes in the physical and psychosocial status of individuals nearing the end of their seventh decade of life—the beginning of senescence—have been inadequately described.

To evaluate the predictors of mortality, morbidity, and other physical/mental outcomes among younger elderly Japanese, we established an age-specific, prospective cohort that focused on people who would soon reach 65 years of age. We then followed them until they passed their 70th birthday. Herein, we describe the study design and the profiles of cohort participants at baseline. This study has been designated the New Integrated Suburban Seniority Investigation Project (The NISSIN Project).

## METHODS

### Setting and participants

In 1994, researchers affiliated with the former Department of Preventive Medicine, Nagoya University School of Medicine, designed a community-based, prospective cohort study that was combined with preventive health services in Nissin City, which is located in central Japan near metropolitan Nagoya. Nissin has an area of 34.0 km^2^ and a population of approximately 60 000 in 1995. Its mean annual population growth rate from 1995 through 2000 was the highest (16.4%) of any city in Japan.^19^ Because its government has been committed to conducting welfare projects for elderly inhabitants, Nissin was selected as the most suitable community for conducting a study on aging. We launched the prospective cohort in 1996 as part of preventive health services offered by the municipal government.

Using the basic resident registry of the city, residents aged 64 years were invited by letter to a free comprehensive medical and dental check-up in April every year from 1996 through 2005, and the responders were registered as our cohort members. The medical check-up included height and weight; systolic and diastolic blood pressure; dipstick urinalyses; fecal occult blood; laboratory blood testing including blood counting, liver function, serum lipids, and serum creatinine; chest x-ray; upper gastrointestinal fluorography; electrocardiography; and (in women) measurement of calcaneal bone density using dual energy X-ray absorptiometry (through 2004) or dry ultrasonometry (since 2005). All clinical tests were performed at a single laboratory. Concurrently, several mini-tubes (100–500 µL per tube) of plasma from each examinee were stored for future analyses at −80 °C in freezers at the Department of Preventive Medicine/Biostatistics and Medical Decision Making, Nagoya University Graduate School of Medicine. The self-administered questionnaire included items on demographic and lifestyle factors, physical function, and quality of life. Demographic factors included sex and year of birth; residential, marital, educational, and work status; source of income; and current history of diseases and medication. Lifestyle factors included smoking status, alcohol consumption, sleep quality, leisure time activities, physical activities, eating habits, and diet. Oral hygiene habits, including frequency of brushing, were included in the questionnaire, and the number of teeth lost (excluding third molars), use of dentures, and periodontal status were noted at the dental examination.

In the section on diet, the questionnaire included items on the frequency of intake for 97 modern Japanese foods and dishes during the 1 month before the baseline survey. This food frequency questionnaire was designed to estimate daily amounts of food group consumption and nutrient intakes, and has been previously validated.^20^^,^^21^ Intellectual, physical, and social functioning were evaluated by using the Tokyo Metropolitan Institute of Gerontology (TMIG) index of competence—a standardized, multidimensional, 13-item index of competence that measures the functional capacity of elderly individuals and has been adapted for and validated in the Japanese population.^22^^,^^23^ Quality of life was measured by using the Life Satisfaction Index-K and the shorter version of the Geriatric Depression Scale, with 15 items. The Life Satisfaction Index-K questionnaire measures subjective well-being among elderly individuals and consists of 9 items (1 item from the Kutner Morale Scale,^24^ 5 items from the revision of the Philadelphia Geriatric Center Morale Scale,^25^ and 3 items from the Life Satisfaction Index-A^26^). The questionnaire has been demonstrated to have sufficient reliability and validity in the Japanese population.^27^ The Geriatric Depression Scale is a self-rating depression screening tool for elderly individuals^28^^,^^29^; the shorter version has been validated.^30^^–^^32^ In other examinations, the participants underwent physical fitness testing, including sitting trunk flexion; right and left grip strength; one-leg standing with eyes open (from 1996 through 2002); gait performance testing, including step length, pitch, and velocity during comfortable and maximum walking (from 1996 through 2002); cognitive function testing, including the word recall and the delayed word recall task from the Alzheimer’s Disease Assessment Scale (ADAS)^33^ and the forward and backward Digit Span Test from Wechsler Adult Intelligence Scale-Revised (WAIS-R)^34^ (from 1996 through 2001); and a salivary IgA test (from 1998 through 2005).

### Follow-up

Since 2002, we have been conducting annual health checkups similar to those conducted at baseline for city residents aged 70 years. Clinical and laboratory tests of the same items, the cognitive function test (ADAS), and the short questionnaire, which includes an item on the frequency of falls, were offered. The study participants who still resided in the city were able to participate in these health checkups and their study outcomes were then obtained from these measurements. For those who lived in the city but did not participate in these health checkups, at-home interviews were conducted by municipal public health nurses. By using the basic resident register of the city, participant mortality and relocation out of the city were followed. The cause of death was confirmed by using the official death certificate, with the permission of the Ministry of Internal Affairs and Communications of Japan. Visiting municipal public health nurses noted whether cohort members received home nursing care under the public nursing-care insurance system.

### Ethical issues

Eligible respondents gave consent to participate in the study at the site of the health check-up. For the questionnaire survey, oral consent was obtained by using an opt-out approach up to 2001, and written consent by an opt-in approach thereafter.^35^ Similarly, for blood sample donation, oral consent was obtained by the opt-out approach up to 1999, and written consent by the opt-in approach thereafter. The study protocol was approved by the Ethics Committee of Nagoya University Graduate School of Medicine (No. 162, 2002 and 2004), the National Center for Geriatrics and Gerontology of Japan (No. 242, 2006), and the Aichi Medical University School of Medicine (No. 558, 2008).

### Data analyses

All data were anonymized, and data cleaning procedures were rigorously conducted by one of the authors (AT). We tabulated the numbers of the participants, along with their proportions and the mean values of measurements, for selected baseline characteristics, by sex. Body mass index was calculated as weight (kg) divided by height (m) squared, and obesity was defined as a BMI of 25.0 kg/m^2^ or higher. Hypertension was defined as a measured systolic blood pressure of 140 mm Hg or higher, a diastolic blood pressure of 90 mm Hg or higher, or self-reported medication for hypertension. Diabetes mellitus was defined as a measured hemoglobin A1c of 6.5% or higher, fasting plasma glucose of 126 mg/dl or higher, or self-reported medication for diabetes mellitus. Hyperlipemia was defined as a total cholesterol level of 220 mg/dl or higher, or self-reported medication for hyperlipemia. Glomerular filtration rate (GFR) was estimated by the Modification of Diet in Renal Disease (MDRD) study equation, modified for Japanese.^36^ According to the guidelines of the Kidney Disease Outcomes Quality Initiative, GFR less than 60 mL/min/1.73 m^2^ represents stage 3 to 5 chronic kidney disease.^37^ Cerebrovascular diseases indicated cerebral infarction, cerebral hemorrhage, and subarachnoid hemorrhage; cardiovascular diseases indicated myocardial infarction and angina pectoris; and cancers included solid tumors of the lung, stomach, gall bladder, ovary, uterus, breast, small intestine, colorectum, and liver. We excluded participants with log_e_-transformed energy intake levels beyond 3 standard deviations from the mean value per day by sex from the analyses for nutrition.^38^ The possible total score range in the LSI-K is 0 to 9, and a higher score indicates greater life satisfaction.^27^ The possible total score range in the TMIG index of competence is 0 to 13, and a higher score indicates a greater competence level.^22^^,^^23^ Although the total GDS-15 score ranges from 0 to 15 and a higher score suggests more severe depression, a total score of 6 or higher was treated as significant depressive tendency.^30^^–^^32^ Sex differences were evaluated using the unpaired *t*-test for numerical variables, and the chi-square test for categorical variables. All statistical analyses were carried out using SPSS software (version 16.0J, SPSS, Inc.).

## RESULTS

Table 1 shows the temporal trend in participation for the study. The number of eligible residents aged 64 years increased from 540 in 1996 to 903 in 2005, and totaled 7004. Among the eligible subjects, 3098 participated in the medical check-up, and 3073 participants (43.9%) were ultimately enrolled in the study, excluding 25 persons who did not provide consent. Nearby half of the eligible residents participated in the study, but the proportion decreased after 2002. A total of 1548 men and 1525 women were enrolled in the study—a sex ratio of approximately 1.0. Among the participants, 2608 (84.9%) underwent a dental examination, 1147 (37.3%) a cognitive function test, 1349 (43.9%) a fitness test, and 2472 (80.4%) a salivary IgA test.

**Table 1.** Temporal trend in participation for the study

		Eligible	Participants			Response
Entry year	Year of birth	residents	Men	Women	Total	rate
		
		*n*	*n*	*n*	*n*	%
1996	1931	540	124	129	253	46.9
1997	1932	612	131	134	265	43.3
1998	1933	593	131	137	268	45.2
1999	1934	637	152	169	321	50.4
2000	1935	679	165	158	323	47.6
2001	1936	718	208	158	366	51.0
2002	1937	823	173	154	327	39.7
2003	1338	720	160	173	333	46.3
2004	1939	779	142	144	286	36.7
2005	1940	903	162	169	331	36.7

Total		7004	1548	1525	3073	43.9

Table 2 shows the baseline demographic and lifestyle characteristics of the study participants, by sex. The proportions of current workers, presently married individuals, and high academic achievers were greater among men than among women; the proportions of current smokers and drinkers were markedly greater among men than among women.

**Table 2.** Selected baseline demographic and lifestyle characteristics of participants, by sex

	Men (*n* = 1548)		Women (*n* = 1525)		*P* value
		
	*n*	%	*n*	%	
Demographic status					
​ Currently employed	863	55.7	399	26.2	<0.001
​ Presently married	1469	94.9	1254	82.2	<0.001
​ College graduate	451	29.1	62	4.1	<0.001

Lifestyle factors					
​ Current smoker	376	24.3	45	3.0	<0.001
​ Ex-smoker	502	32.4	52	3.4	<0.001
​ Current drinker	1061	68.5	298	19.5	<0.001

Table 3 shows the medical and dental characteristics of the study participants, by sex. Obesity, hypertension, diabetes mellitus, and stage 3 to 5 chronic kidney disease were more common in men than in women. However, the prevalence of hyperlipemia was lower in men than in women. A history of cardiovascular diseases and a history of cerebrovascular diseases were more frequent in men than in women. The prevalence of cancers was similar between sexes. The frequency of tooth brushing at least 3 times per day was higher in women than in men. Approximately 75% of the male and female participants had 20 teeth or more.

**Table 3.** Selected baseline medical and dental characteristics of participants, by sex

	Men		Women		*P* value
	
	*n*	%	*n*	%	
Medical status					
​ Obesity^a^	384	24.8	306	20.1	<0.001
​ Hypertension^b^	781	50.5	599	39.3	0.002
​ Diabetes mellitus^c^	197	12.8	95	6.2	<0.001
​ Hyperlipemia^d^	499	32.3	894	58.6	<0.001
​ Chronic kidney disease ​ (stage 3–5)^e^	385	24.9	315	20.7	0.006
​ History of					
​ ​ Cardiovascular diseases	77	5.0	36	2.4	<0.001
​ ​ Cerebrovascular diseases	62	4.0	39	2.6	0.026
​ ​ Cancers	40	2.6	36	2.4	0.729

Dental status					
​ Brushing ≥ 3 times/day	165	12.7	259	20.7	<0.001
​ Number of teeth present ≥ 20	896	77.4	849	75.3	0.323

^a^Body mass index ≥ 25.0 kg/m^2^.

^b^Systolic blood pressure ≥ 140 mm Hg, diastolic blood pressure ≥ 90 mm Hg, or medication for hypertension.

^c^Hemoglobin A1c ≥ 6.5%, fasting plasma glucose ≥ 126 mg/dl, or medication for diabetes mellitus.

^d^Total cholesterol ≥ 220 mg/dl or medication for hyperlipemia.

^e^GFR ≤ 60.0 ml/min/1.73 m^2^, as estimated by the MDRD study equation, modified for Japanese.

Table 4 shows dietary intakes of representative foods and nutrients among the study participants. Although energy intake was similar between sexes, intake of all nutrients was lower in men than in women, except for alcohol. Table 5 shows the baseline aging-related psychosocial state of the study participants. The scores were similar between sexes, but the proportion with depressive tendency was slightly smaller in men than in women. Both male and female participants had very high scores in the TMIG index of competence.

**Table 4.** Selected baseline nutritional characteristics of participants, by sex^a^

	Men (*n* = 1481)		Women (*n* = 1460)		*P* value
	
	Mean	SD^c^	Mean	SD^c^	
Energy (kcal)^b^	1910.5	601.7	1910.3	611.6	0.804
Fat (g)	50.1	21.7	57.2	24.0	<0.001
Protein (g)	69.1	26.0	77.6	29.5	<0.001
Carbohydrate (g)	256.8	87.3	261.2	82.3	0.168
Meat (g)	48.6	36.4	58.0	44.6	<0.001
Fish (g)	71.5	51.2	86.1	61.0	<0.001
All vegetables (g)	216.2	129.4	276.7	151.8	<0.001
​ Green-yellow ​ vegetables (g)	103.3	92.5	137.3	103.1	<0.001
​ Other vegetables (g)	112.6	61.4	139.0	76.1	<0.001
Fruit (g)	156.7	115.1	249.2	157.5	<0.001
Alcohol (g)	23.5	19.6	4.3	11.3	<0.001

^a^Daily intake of energy and each nutrient was estimated from a food frequency questionnaire.

^b^Ten men and 10 women with log_e_-transformed energy intake values greater than 3 standard deviations from the mean energy intake per day by sex were excluded from the analyses for nutrition.

^c^Standard Deviation.

**Table 5.** Selected baseline psychosocial characteristics of participants, by sex

	Men		Women		*P* value
TMIG^a^ index ​ of competence, *n*	1542		1516		
​ Median (IQR^b^)	12	(11–13)	13	(12–13)	<0.001

Life Satisfaction Index-K, *n*	1537		1514		
​ Median (IQR)	5	(4–7)	5	(4–7)	0.930

Geriatric Depression ​ Scale-15, *n*	1538		1513		
​ Median (IQR)	3	(1–5)	4	(2–5)	<0.001
​ Depressive tendency ​ (total score ≥ 6), *n* (%)	310	(20.2)	363	(24.0)	0.018

^a^Tokyo Metropolitan Institute of Gerontology.

^b^Interquartile range.

## DISCUSSION

To our knowledge, this is the first report of an age-specific, prospective cohort among a younger elderly population. Herein, we described the rationale and study design, and briefly presented the baseline demographic, lifestyle, clinical, and psychological characteristics of participants reaching the early-senescence age of 65 years.

Over the past 50 years, many cohort studies on aging and its related factors have been conducted in Japan and in western countries.^13^^–^^12^ Although these studies have evaluated various risk factors relevant to mortality and morbidity, in addition to changes in physical, psychological, and cognitive functions, the participants of these studies were people aged 70 years or older,^7^^,^^9^^,^^11^^,^^13^^,^^14^ or were of widely dispersed ages.^10^^,^^15^^,^^16^^,^^18^ The life table of the Japanese population (data available through the Internet, http://www.mhlw.go.jp/toukei/saikin/hw/life/20th/index.html)^39^ shows that the death rate markedly accelerates after the age of 65. Thus, cohorts of older elderly people would be selectively composed of survivors of early senescence. In addition, detailed physical and psychological examinations would be overly challenging for the older elderly because of their relatively advanced functional deterioration. On the other hand, in cohorts comprising people aged 40 or 50 years, significant health events would be rare during the early follow-up period, resulting in a lack of statistical power. Another point to consider is that most Japanese employees retire between the age of 60 and 65, and their lifestyles dramatically change at this time. Our cohort allows us to investigate participant lifestyles and clinical or subclinical physical/psychosocial status at the middle of their seventh decade of life. These factors are likely to be important predictors of subsequent enduring physical and mental well-being.

Our cohort is rather unusual because it is composed of a uniform age-specific group of people. Age is one of the most important factors that affect health events. In epidemiological studies, therefore, multivariable analyses are performed that treat age as a covariate. However, the manner of age attribution is not necessarily linear, and some unadjusted age effects may remain. Our age-specific study design completely eliminates age-derived biases. To our knowledge, there are 2 other age-specific cohort studies of elderly people in Japan: the Koganei Study^7^ enrolled people aged 70 years as the study participants, and a study conducted by Kyushu Dental College^9^ registered elderly people aged 80 years as cohort members. Their response rates (43.5% and 54.4%, respectively) and measurements of lifestyle, activity level, quality of life, and physical and cognitive function were similar to those of our project; however, the target ages were higher. Our cohort comprises more than 3000 participants and is larger than other elderly cohorts, which should ensure the validity of our analyses. Moreover, this cohort is characterized by an annual 6-year consecutive entry–exit system. Every year, several hundred 64-year-olds enter the cohort as a similar number leave it. We therefore were able to invest less manpower in registration and baseline/outcome measurements than is necessary for the simultaneous entry system that is commonly used in community-based cohort studies.

Some study limitations warrant consideration. First, although our study was combined with a free medical check-up, the response rate was not high (43.9%). In general, some differences in characteristics are found between respondents and nonrespondents. Elderly respondents are reported to be subjectively healthier and more satisfied with life than nonrespondents.^40^ Socioeconomic status might confound with participation in health checkups.^41^ Although we were unable to obtain any data on the health status of nonrespondents, our study participants, because they were respondents, probably had better health-related characteristics than did nonrespondents. To assess the representativeness of the participants in our cohort, we compared the participants’ characteristics with data from several available nationwide surveys: the National Health and Nutrition Survey in Japan, 2004 (data available through the Internet, http://www.mhlw.go.jp/houdou/2006/05/h0508-1a.html),^42^ and the National Survey of Dental Disease in Japan, 2005 (data available through the Internet, http://www.mhlw.go.jp/topics/2007/01/tp0129-1.html).^43^ The condition of the present study participants was somewhat better than that of the general Japanese population aged 60 to 69 years (Table 6). The frequencies of smoking, alcohol consumption, obesity, and hypertension, for example, were slightly lower in the cohort members than in the general population, whereas that of hyperlipemia was higher in the cohort members. Although a low response rate might yield a non-negligible selection bias in descriptive epidemiology, the effects of such bias would be rather small in association analyses between outcomes and their risk factors in a cohort. Nevertheless, this possible bias should be taken into consideration when generalizing the study results.

**Table 6.** Selected health-related characteristics in general Japanese population, by sex (summarized from data from nationwide surveys)

	Age 60–69	

	Men	Women
Lifestyle^a^		
​ Current smoker (%)	33.3	7.6
​ Current drinker (%)	73.0	29.4

Medical status^a^		
​ Obesity (%)^c^	29.7	29.9
​ Hypertension (%)^d^	67.7	55.9
​ Hyperlipemia (%)^e^	28.7	49.6

Dental status^b^		
​ Brushing ≥ 3 times/day (%)	15.2	27.5
​ Number of teeth present ≥ 20 (%)	65.3	61.7

Nutritional status^a^		
​ Energy intake (kcal)	2182.0	1769.0
​ Fat (g)	53.4	47.0
​ Protein (g)	81.4	69.3
​ Carbohydrate (g)	306.3	260.1
​ Meat (g)	68.9	53.7
​ Fish (g)	124.1	95.1
​ All vegetables (g)	312.4	295.9
​ Fruits (g)	151.9	177.8

^a^National Health and Nutrition Survey in Japan, 2004.

^b^Survey of Dental Disease, 2005.

^c^Body mass index ≥ 25.0 kg/m^2^.

^d^Systolic blood pressure ≥ 140 mm Hg, diastolic blood pressure ≥ 90 mm Hg.

^e^Total cholesterol ≥ 220 mg/dl.

Because we annually recruited the study participants from 1996 through 2005, our observations might have been affected by the passage of time.^44^ Issues related to this time trend will be addressed in a future article.

As of April 2009, the enrollment of cohort members has been completed and follow-up health checks are ongoing. The NISSIN Project is a promising way to collect valuable information on well-being in this new era of longevity.
